# Clinical application of ultrasound-guided Core Needle Biopsy Histology and Fine Needle Aspiration Cytology in Cervical Lymph Nodes

**DOI:** 10.12669/pjms.39.3.6630

**Published:** 2023

**Authors:** Wei-na Mu, Jian-heng Li, Ying Liu, Ying Wen, Xin Liu

**Affiliations:** 1 Wei-na Mu, Department of Ultrasound, Baoding No.1 Central Hospital, Baoding 071000, Hebei, China; 2 Jian-heng Li, Hebei University, Baoding 071000, Hebei, China; 3 Ying Liu Department of Ultrasound, Baoding Baoshihua Oriental Hospital, Baoding 071051, Hebei, China; 4 Ying Wen Department of Ultrasound, The 82^nd^ Group Army Hospital of the People’s Liberation Army of China, Baoding 071000, Hebei, China; 5 Xin Liu, Department of Ultrasound, Baoding No.1 Central Hospital, Baoding 071000, Hebei, China

**Keywords:** Cervical lymph nodes, Core needle biopsy histology, Fine needle aspiration cytology, Ultrasound guidance, Complication

## Abstract

**Objectives:**

To investigate the difference of application of core needle biopsy histology and fine needle aspiration cytology in cervical lymphadenopathy.

**Methods:**

A retrospective analysis was made on 80 patients with cervical lymphadenopathy admitted to Baoding No.1 Central Hospital from to October 2018 to February 2020, and they were randomly divided into two groups: core needle group and fine needle group. Patients in the core needle group were given core needle biopsy histology, while those in the fine needle group were given fine needle aspiration cytology, and the puncture results and surgical complications were compared between the two groups.

**Results:**

The accuracy rates of the core needle group and the fine needle group in the diagnosis of malignant cervical lymph nodes were 95.83% and 72.22% respectively, with a statistically significant difference (*χ²*=4.683, *p=*0.030). The sensitivity, specificity, positive predictive value and negative predictive value of the core needle group were 100.00%, 93.75%, 95.83% and 100.00% respectively, while those of the fine needle group were 86.67%, 90.00%, 86.67% and 90.00% respectively, with no statistically significant differences between the two groups (*p>*0.05). The complication rate in the core needle group was 22.50%, which was higher than the 5.00% in the fine needle group (*χ²*=5.165, *p=*0.023).

**Conclusions:**

No significant difference was observed between core needle biopsy histology and fine needle aspiration cytology in diagnosing cervical lymphadenopathy, but the former has a high complication rate.

## INTRODUCTION

Lymphatic system is an important component part of human body’s important immune and defense system, and lymph nodes are the dominant ones. Among the numerous lymph nodes, cervical lymph node accounts for 1/3 of the whole body lymphatic system.^1^,^2^ It is found that cervical lymphadenopathy is a common clinical disease and the common location of lymphadenopathy.^3^,^4^ Given that lymphadenopathy is the reflection of the lymphatic system’s response to various pathogenic factors and tumor cells, it is crucial to correctly judge the benign and malignant nature of cervical lymphadenopathy for the diagnosis and treatment of the disease. Despite the role of high-frequency color Doppler ultrasound in identifying the nature of lymph node lesions, it cannot provide pathological diagnosis.^5^ If pathological examination of lymph nodes is carried out, a lymph node biopsy is required to determine the pathological types of lymph node lesions. Ultrasound-guided puncture biopsy is currently recognized as one of the effective means for the diagnosis of lymph node lesions, boasting various advantages such as simple and easy operation, accurate target, less trauma and less bleeding under the guidance of ultrasound.^6^ As for puncture, it is divided into fine needle aspiration cytology and core needle biopsy histology.^7^,^8^ However, the two methods are different in safety and accuracy.^9^ To this end, fine needle and core needle were used for puncture biopsy respectively in our hospital to compare the differences between the two techniques.

Our objective was to investigate the difference of application of core needle biopsy histology and fine needle aspiration cytology in cervical lymphadenopathy

## METHODS

A retrospective analysis was made on 80 patients with cervical lymphadenopathy admitted to our hospital from to October 2018 to February 2020. The study was approved by the Institutional Ethics Committee of Baoding First Central Hospital on March 1^st^, 2021 (No.:[2021-011]), and written informed consent was obtained from all participants.

### Inclusion criteria:

- Patients with complete clinical data;
- Patients with valid pathological diagnosis report issued by the department of pathology;
- Patients who have informed consent and signed the consent form;
- Patients with good mental state and willing to actively cooperate with this study.

### Exclusion criteria:

- Patients with contraindication of puncture;
- Patients with abnormal coagulation function;
- Patients with disturbance of consciousness and mental illness.

All 80 patients were divided into two groups by random number method: core needle group and fine needle group, with 40 cases in each group. No statistically significant differences were observed between the two groups in terms of gender, age and lymph node location (*p>*0.05). See Table-I.

**Table-I:** Comparison of general data between the two groups.

Item	Core needle group (n=40)	Fine needle group (n=40)	t/² value	p value
Age (years old)	45-73 (56.18±6.81)	42-73 (57.08±7.18)	0.575	0.567
Gender (male/female, n)	24/16	22/18	0.205	0.651
Lymph node location			0.675	0.713
Zone II	12	9		
Zone III	20	21		
Zone IV	8	10		

The diseased lymph nodes were examined by color Doppler ultrasonography, and the elasticity button was pressed to judge its softness and hardness. After that, the largest section of the lesion or the section with the richest blood flow display was selected, and the contrast mode was switched. In contrast mode, the mechanical index (MI) was set at 0.06-0.14, and 2.4 ml of contrast agent was injected into the patient’s cubital vein by bolus. Meanwhile, a timer was started, and the position of the probe was fixed to keep the section unchanged to continuously observe the dynamic perfusion process of the lesion in real time.

According to the location of lymph nodes of patients and the results of ultrasound examination, the target lymph nodes and the target area were selected to determine the optimal puncture location and puncture path. Then disinfection and towel-laying were carried out, and 1% lidocaine was used for local infiltration anesthesia. In the fine needle group, 22G fine needle was used for negative pressure puncture biopsy. Under the ultrasonic probe, the needle tip was inserted into the center of the target area, and the negative pressure state was kept and the suction was repeated for 10-15 times at different angles. The extract was smeared, fixed with 95% ethanol, and sent for pathological examination. In the core needle group, 18G core needle was used for biopsy. Under the guidance of ultrasonic probe, the puncture needle was sent to the target area. After that, the puncture needle was quickly withdrawn, the cut lesion tissue strips were taken out and fixed with 10% formaldehyde, and then sent for pathological examination.

### Observation indicators:

The puncture results of the two groups were observed, and their sensitivity, specificity, positive predictive value and negative predictive value of the examination results were compared. Furthermore, the complications of the two groups, including bleeding, fever, abscess formation, sinus formation and pain aggravation, were observed.

### Statistical Analysis:

All the data in this study were statistically analyzed by SPSS 22.0. The measurement data were expressed by mean standard deviation (*x̅*±*S*), and the comparison between groups was made by *t*-test; The enumeration data were expressed as *n* (%), and the *χ²* test was used for comparison between groups. P*<*0.05 indicates a statistically significant difference.

## RESULTS

There was no significant difference in the accuracy of fine needle group and the core needle group in diagnosing benign cervical lymph nodes (*p>*0.05). The accuracy of the fine needle group and the core needle group in diagnosing malignant cervical lymph nodes was 95.83% and 72.22% respectively, with a statistically significant difference (*χ²*=4.683, *p=*0.030), see Table-II and Table-III.

**Table-II:** Comparison of core needle biopsy results and pathological results[*n* (%)].

Pathological nature (n=40)	Core needle biopsy results			Total

	Benign	Malignant	Uncertainty	
Benign	15 (93.75)	1 (6.25)	0 (0.00)	16 (40.00)
Malignant	0 (0.00)	23 (95.83)	1 (4.17)	24 (60.00)

**Table-III:** Comparison of core needle biopsy results and pathological results[*n* (%)].

Pathological nature (n=40)	Fine needle aspiration results			Total

	Benign	Malignant	Uncertainty	
Benign	18 (81.82)	2 (9.09)	2 (9.09)	22 (55.00)
Malignant	2 (11.11)	13 (72.22)	3 (16.67)	18 (45.00)

No statistically significant differences were observed in the diagnostic sensitivity, specificity, positive predictive value and negative predictive value of the two examination methods (*p>*0.05), see Table-IV.

**Table-IV:** Comparison of sensitivity, specificity, positive predictive value and negative predictive value between the two groups.

Group	Sensitivity (%)	Specificity (%)	Positive predictive value (%)	Negative predictive value (%)
Core needle group	100.00	93.75	95.83	100.00
Fine needle group	86.67	90.00	86.67	90.00
c^2^	3.237	0.164	1.092	1.591
p	0.072	0.686	0.296	0.207

The complication rate of patients in the core needle group was 22.50%, which was significantly higher than 5.00% in the fine needle group, with a statistically significant difference (*p<*0.05). Table-V.

**Table-V:** Comparison of surgical complications between the two groups[n, (%)].

Group	n	Bleeding	Fever	Abscess formation	Sinus formation	Aggravated pain	Subtotal
Core needle group	40	4 (10.00)	2 (5.00)	1 (2.50)	0 (0.00)	2 (5.00)	9 (22.50)
Fine needle group	40	1 (2.50)	1 (2.50)	0 (0.00)	0 (0.00)	0 (0.00)	2 (5.00)
*χ^2^* value							5.165
p value							0.023

## DISCUSSION

It was reported by Ternov et al.^10^ that ultrasound-guided core needle biopsy can diagnose 89.6% of cervical lymph nodes and distinguish their subtypes, while the result in this study was 95.83%, which was slightly higher than the above study. In this study, only one case showed that the necrotic tissue failed to make a definite diagnosis, which may be due to the necrotic area or the sampling site. One case of benign cervical lymphadenopathy was misdiagnosed as malignant, and postoperative pathology showed that some cells were heteromorphic, which may be related to excessive extrusion and deformation of tissues and gene overlap in lymphocytes during puncture. In this study, five cases of fine needle cytology biopsy failed to make a definite diagnosis, four cases showed necrotic tissue due to insufficient materials, and one case showed necrotic tissue. Four cases were misdiagnosed; By analyzing the reasons, we consider it is mainly related to insufficient materials, and it is difficult for fine needles to suck enough diseased cells. Therefore, before the ultrasound-guided core needle biopsy, the lymph nodes can be examined by contrast-enhanced ultrasound, the size and necrotic area of lymph nodes can be defined, and multi-point and multi-directional sampling can be performed to improve the sampling quality. When fine needle aspiration cytology is performed, a large number of tissue cells should be aspirated as much as possible in multiple directions, so as to improve the diagnostic rate and reduce misdiagnosis.

Cervical lymph nodes are an important component part of the lymphatic system of the whole body, and the lymph of the whole body can be drained to the cervical lymph node.^11^,^12^ Cervical lymphadenopathy is the metastatic position of malignant tumors such as esophageal cancer, lung cancer and thyroid cancer, and the lymph nodes also have their own abnormal lesions, such as necrotizing lymphadenitis and lymphoma.^13^ How to accurately diagnose the benign and malignant cervical lymphadenopathy is of great significance for the early diagnosis of the disease, which can provide reliable basis for treatment and improve the survival rate of patients.^14^,^15^ Up to now, the gold standard for the diagnosis of cervical lymphadenopathy is still the pathological examination of lymph nodes after surgical resection.^16^,^17^ With the development of ultrasound technology, ultrasound-guided puncture biopsy has become popular. Ultrasound-guided puncture is simple, accurate in location, without special preparation before puncture, and this method has a wide application range.

Pathological types of cervical lymph nodes can be divided into three types: malignant metastatic lymph nodes, lymphoma and benign lymphadenopathy, among which malignant metastatic lymph nodes are the most common. In this study, malignant lymph nodes in both groups account for a large proportion.

Fine needle aspiration cytology has the advantages of simple operation, little trauma, safety and high diagnostic accuracy.^18^,^19^ In this study, the diagnostic accuracy of fine needle aspiration cytology for cervical swollen lymph nodes was 81.82% and 72.22% respectively, and the study confirmed that fine needle aspiration cytology could obtain satisfactory cytopathological results. However, the sensitivity and specificity of the target lymph node location, size and puncture level are relatively low.^20^ In our hospital, histological examination of core needle puncture can take out complete tissue, so its accuracy, sensitivity and specificity are high. After analyzing the reasons, we think that there are the following possible reasons: (1) The core needle is clearer in ultrasonic images, so it is easy to guide puncture; (2) The core needle is selected by biopsy gun, which is less affected by the operator’s experience, while the fine needle requires higher technical level of the operator; (3) Fine needles collect mainly liquid components, while thick needles collect solid tissue strips, so it is easy for naked eyes to judge whether the specimen meets the requirements. In this study, there was no significant difference in sensitivity, specificity, positive predictive value and negative predictive value between the two groups of patients (*p>*0.05). The analysis of the reasons is mainly related to the small sample size, so it is necessary to further increase the sample size. But core needle puncture has the disadvantage of high complication rate. Moreover, the core needle puncture was easy to cause tumor cells to be planted in the needle channel, leading to tumor spread. The conclusions of this study supplement clinical research data and reference data for the selection of two puncture methods.

### Limitations of this study:

This study was a retrospective descriptive study, with limited clinical data available and limited persuasive conclusions. Further intervention trials are needed in the future to confirm these results.

## CONCLUSION

No significant difference was observed between core needle biopsy histology and fine needle aspiration cytology in diagnosing cervical lymphadenopathy, but the former has a high complication rate. In view of this, clinicians can choose different puncture inspection methods according to specific conditions.

### Authors’ Contributions:

**Wei-na Mu** and **Xin Liu** designed this study and prepared this manuscript,and are responsible and accountable for the accuracy and integrity of the work.

**Jian-heng Li** and **Ying Wen** collected and analyzed clinical data.

**Ying Liu** Data analysis, significantly revised this manuscript.
